# Predictors of Lung Cancer Risk: An Ecological Study Using Mortality and Environmental Data by Municipalities in Italy

**DOI:** 10.3390/ijerph18041896

**Published:** 2021-02-16

**Authors:** Claudio Gariazzo, Alessandra Binazzi, Marco Alfò, Stefania Massari, Massimo Stafoggia, Alessandro Marinaccio

**Affiliations:** 1 Occupational and Environmental Medicine Epidemiology and Hygiene Department, Italian Workers’ Compensation Authority (INAIL), 00144 Rome, Italy; a.binazzi@inail.it (A.B.); s.massari@inail.it (S.M.); a.marinaccio@inail.it (A.M.); 2 Department of Statistic, University of Roma “Sapienza”, 00185 Rome, Italy; marco.alfo@uniroma1.it; 3 Department of Epidemiology, Lazio Regional Health Service, ASL Roma 1, 00154 Rome, Italy; m.stafoggia@deplazio.it

**Keywords:** asbestos, COPD, ischemic heart, PM_2.5_, radon, urbanization, deprivation, occupational respiratory diseases

## Abstract

Lung cancer (LC) mortality remains a consistent part of the total deaths occurring worldwide. Its etiology is complex as it involves multifactorial components. This work aims in providing an epidemiological assessment on occupational and environmental factors associated to LC risk by means of an ecological study involving the 8092 Italian municipalities for the period 2006–2015. We consider mortality data from mesothelioma as proxy of asbestos exposure, as well as PM_2.5_ and radon levels as a proxy of environmental origin. The compensated cases for occupational respiratory diseases, urbanization and deprivation were included as predictors. We used a negative binomial distribution for the response, with analysis stratified by gender. We estimated that asbestos is responsible for about 1.1% (95% CI: 0.8, 1.4) and 0.5% (95% CI: 0.2, 0.8) of LC mortality in males and females, respectively. The corresponding figures are 14.0% (95% CI: 12.5, 15.7) and 16.3% (95% CI: 16.2, 16.3) for PM_2.5_ exposure, and 3.9% (95% CI: 3.5, 4.2) and 1.6% (95% CI: 1.4, 1.7) for radon exposure. The assessment of determinants contribution to observed LC deaths is crucial for improving awareness of its origin, leading to increase the equity of the welfare system.

## 1. Introduction

In 2018, 1,761,007 lung cancer deaths have been recorded worldwide (18.4% of the total deaths). Age standardized incidence and mortality rates (per 100,000) were 22.5 and 18.6, respectively [1]. Lung cancer risk can be associated with exposure to pollutants originating from working and environment-related conditions, lifestyles, smoking, living places, e.g., urbanization level and access to health care services.

It was repeatedly observed that occupational exposures significantly contribute to lung cancer risk among workers employed in specific industries and job profiles [2]. According to the Global Burden of Disease study, the etiological fraction of lung cancer due to occupational exposure is substantial; 86% of cancer deaths due to occupational carcinogens refer to lung cancer cases, followed by mesothelioma (7.9%) and laryngeal cancer (2.1%) [3].

The most important occupational lung carcinogens are reported to be asbestos, silica, radon, heavy metals, and polycyclic aromatic hydrocarbons [4].

The contribution of asbestos exposure to the occurrence of lung cancer in Europe was estimated to lead to a population attributable risk (PAR) ranging from 10 to 20% among males [5]. In Great Britain, the total excess lung cancer deaths due to asbestos exposure was estimated to be 2–3% of total male lung cancer deaths and its ratio to mesothelioma deaths was between 1:1.5 and 1:1 [6]. Asbestos exposure is mainly linked with work activities, but environmental exposure can be found for those living close to industrial settlements using or processing asbestos or non-professional subjects who haunted places known for presence of asbestos.

In Italy, asbestos has been extensively produced and used, with consumption increasing until the 1980s and declining afterwards [7]. The largest use was in cement production, fireproofing and thermal insulation in shipbuilding and railway carriages. On the basis of CAREX data, the number of Italian asbestos-exposed workers was larger than 350,000 just before the ban in 1992, and dropped to about 76,000 in 2005 [8].

The first study providing, for Italy, indirect estimates of asbestos-related lung cancer cases was conducted in Piemonte region and estimated that 3.9% of lung cancer deaths can be attributed to asbestos [9]. In that case, pleural cancer mortality at municipality level was used as a proxy of exposure to asbestos. This approach was also applied in another ecological study, based on lung and pleural cancer deaths in all Italian municipalities, that estimated a pleural to lung cancer ratio of 1:1 and about 3% (700 cases) of all male lung cancer deaths attributable to asbestos exposure [10].

Radon is another important cause of lung cancer, besides smoking and exposure to occupational carcinogens [11]. The International Agency for Research on Cancer (IARC) classified Radon-222 and its decay products as a known cause of human cancer [1]. The global burden of lung cancer mortality that can be attributed to radon was estimated in 2012 for 66 countries, with a median PAR of 16.5% and a total number of radon-attributable lung cancer deaths of 226,057, representing a median of 3.0% of total cancer deaths [12].

The association of lung cancer mortality with levels of (neighborhood) deprivation has also been investigated A Swedish study provided, for incidence and mortality odds ratio (OR), estimates of 1.27 and 1.32 in the most deprived neighborhoods. Rates of lung cancer were higher among people living in deprived when compared to wealthy neighborhoods. Socioeconomic deprivation has also been linked to incidence of Chronic Obstructive Pulmonary Disease (COPD), a known risk factor for lung cancer, as the degree of airway obstruction is a predictor of lung cancer [13]. Socio-economic deprivation works also through limited access to health services.

Differences between urban and rural areas have also been considered as potential key factors for lung cancer risk, with urban areas often being characterized by higher lung cancer mortality. Plausible explanations lead to factors such as inhabitants’ personal behaviour, air pollution, occupational hazards (more frequent occupational exposure to carcinogens among manual workers—especially males), as well as association between deprivation and smoking. A decreasing mortality gradient, as we move towards lower urbanization, was observed in the most urbanized areas of the Madrid Region, characterized by a high lung cancer mortality, with greater differences in women and for people under 65 years [14]. In the United States, a decrease in lung cancer incidence rates from 2007 to 2016 was observed for both non-metropolitan and metropolitan counties [15].

A considerable body of research suggested a role for particulate matter (PM) as one of the (major) causes of lung cancer [16]. In particular, exposure to PM_2.5_ is associated with an increase in the risk of lung cancer morbidity and mortality, while the association with PM_10_ has been analyzed less frequently and the results are not as solid as for PM_2.5_ [17,18]. The time and spatial patterns of lung cancer deaths attributed to PM_2.5_, at global and country levels, were indeed the focus for several studies. A multi-country study analyzing data from 195 countries, estimated that lung cancer deaths attributed to PM_2.5_ increased worldwide by 16.5% from 1990 to 2015, with about 250,000 lung cancer deaths in 2015 [19].

The association of lung cancer mortality with all the above determinants is often analyzed by considering just a few of them. Some are used as confounders, like deprivation. Lack of exposure data limits both the number of determinants used and the spatial representativeness of the studies. Consequently, there is the need for more comprehensive analyses, which should include the more relevant determinants of lung cancer mortality, providing information not only for metropolitan areas but also for the (less studied) rural ones. In this framework of complexity, the estimation of the impact of lung cancer predictors is a relevant topic for public health and prevention policies.

The aim of this study is to provide an epidemiological assessment on occupational and environmental factors involved in lung cancer risk, based on an ecological analysis by municipalities in Italy. The findings may help in improving the identification of both key factors in the public compensation of lung cancer and prevention policies.

## 2. Materials and Methods

### 2.1. The Study Design

The burden of mortality from malignant neoplasm of trachea, bronchus, and lung (LC hereafter) that can be attributed to selected determinants, was carried out by means of an ecological study involving the 8092 Italian municipalities (Census 2001). The observed death counts from LC has been used to estimate the fraction that can be attributed to a wide set of determinants, by using an appropriate statistical model. Table 1 lists the variables used as determinants of LC mortality. We included environmental and occupational determinants, proxies of smoking habits, deprivation, and urbanization data, all at the municipality level. Such data were collected from 2006 to 2015. Mortality counts and occupational diseases were provided by municipality, year, age classes and gender.

### 2.2. Mortality and Occupational Data

Mortality data were extracted from the Italian National Institute of Statistics (ISTAT in the following) using the Tenth International Classification of Diseases (ICD-10). As for LC mortality, we selected data with ICD-10 codes C33-C34 by municipality, year, gender, and age classes (0–34, 35–64, 65–74, 75–84, and 85+). The latter, along with the Italian resident population in the period 2006–2015 at municipality level (ISTAT), were used to obtain gender and age-specific rates of LC that were necessary to calculate the expected numbers of LC deaths by municipality, year, and gender (through indirect standardization, with Italy as reference standard population).

We further included mesothelioma mortality (ICD-10 codes C45) as a proxy of occupational/environmental exposure to asbestos in the past [9,10], and the number of compensated cases for occupational respiratory diseases (chronic lower respiratory diseases, ICD-10 codes J40-J47, and lung diseases due to external agents ICD-10 codes J60-J67) as a proxy of occupational exposure to carcinogens. The latter were retrieved by the Italian national workers’ compensation authority (INAIL) that receives claims for occupational diseases and provides compensation, after verifying the occupational origin of the disease. It covers about 80% of the Italian labour force.

Mortality for chronic obstructive pulmonary disease (COPD) and other respiratory conditions (ICD-10 codes J40-J44) and ischemic heart diseases (ICD-10 codes I20-I25) were considered as proxies of smoking habits.

All the aforementioned data have been made available by municipality (8092), year (2006–2015) and gender.

### 2.3. Environmental Data

PM_2.5_ and radon concentrations were considered as environmental co-factors for LC mortality according to a large body of literature [12,17,23,24].

PM_2.5_ annual concentrations for each municipality were derived from data developed within the “Big data in Environmental and occupational Epidemiology (BEEP)” project, which aims to provide new evidence about PM-related health effects. Daily mean concentrations of PM_2.5_ were derived from satellite data and elaborated using machine learning algorithms; the entire process is described elsewhere in details [25,26]. To summarize, spatial and spatiotemporal parameters such as satellite-based aerosol optical depth (AOD), land use and meteorological data were collected for each day in 2006–2015 in Italy. A machine learning model was developed to predict daily PM_2.5_ concentrations for each 1 × 1 km grid cell calibrating AOD, land use and meteorological terms to PM_2.5_ monitoring data. Cross-validated R^2^ was 0.81 for PM_2.5_, thus ensuring reliable accuracy properties. Such data were then averaged spatially over each municipality and temporally on a yearly basis.

As for radon exposure, a national map was not available; therefore, we decided to integrate all available information at regional level. Both observed data (field campaigns in schools, houses, and working places) and regional maps, obtained by means of geo-statistical techniques (based on observed data collected by local environmental protection agencies) were used. Missing data at municipal levels were filled in using either literature data provided by the first national field campaign of radon exposure carried out in 1990 by National Research Institutes [20] or Provincial data when available. Such data are highly heterogeneous as they were collected or estimated in different years, places and according to different measurement protocols. Consequently, they can be considered just indicative of the general radon exposure experienced by the population living in a given municipality. To limit the impact of such heterogeneity, we categorized radon values in five different classes (0–50; 51–100; 101–150; 151–300; 300 + Bq/m^3^).

### 2.4. Geographical Data

We considered the urbanization and deprivation level for each municipality as geographical co-factors involved in the occurrence of LC mortality. With regards to the former (urbanization) we used the following urban classification in 5-levels (low; low-medium; medium; medium-high; high) [21]. Each municipality has been classified according to four parameters: resident population (Census 2001 data), “light-at-night” (“VIIRS” 2015 satellite data), percentage of built areas (Corine 2012 Land Cover database), and density of high traffic roads (TeleAtlas TomTom 2012 road network). These variables were then combined to produce a quantitative urbanization score for each municipality.

As for the latter (deprivation), we used data provided by a National study [22] based on census data about (low level of) education, unemployment, non-home ownership, one parent family and overcrowding, to derive a multidimensional index of social and material deprivation. Combining these indicators, the authors derived a classification of municipalities in five clusters (very deprived, deprived, medium, rich, and very rich).

Both urbanization and deprivation levels were considered as not varying over the entire period of analysis (2006–2015).

Population data (source: ISTAT) were retrieved by year, municipality, gender, and age class, and used to calculate the crude rates and the expected number of LC deaths, through indirect standardization, as specified above.

### 2.5. Statistical Analysis

A generalized linear model (GLM) with negative binomial distribution for the observed response (LC death counts) has been used to estimate the association between LC mortality data at municipal level and a list of potential predictors. The following model was used:

$$\begin{array}{ll} \log\left(\frac{O_{it}}{E_{it}}\right)=\beta_{0}+ & \beta_{1}Year_{t}+\beta_{2}{\mathrm{TMesothelioma}}_{it}+\gamma_{2}{\bar{\mathrm{TMesothelioma}}}_{i}+\beta_{3}{\mathrm{TOccupResp}}_{it}+\gamma_{3}{\bar{\mathrm{TOccupResp}}}_{i}+\beta_{4}pm2.5_{it}\\ & +\gamma_{4}{\bar{pm2.5}}_{i}+\beta_{5}{\mathrm{TCopd}}_{it}+\gamma_{5}{\bar{\mathrm{TCopd}}}_{i}+\beta_{6}{\mathrm{TIschemHeart}}_{it}+\gamma_{6}{\bar{\mathrm{TIschemHeart}}}_{i}+\gamma_{7}Radon_{i}\\ & +\gamma_{8}Urbanization_{i}+\gamma_{9}Deprivation_{i} \end{array}$$

where i denotes municipality, O and E are the observed and expected LC mortality counts, TMesothelioma, TOccupResp, TCopd, TIschemHeart and $\bar{\mathrm{TMesothelioma}}$, $\bar{\mathrm{TOccupResp}}$, $\bar{\mathrm{TCopd}}\;$and $\bar{\mathrm{TIschemHeart}}$ are respectively the yearly and the mean values (calculated over the period at municipal level) for the mortality/claim rates (per 1000 residents) due to mesothelioma, occupational respiratory disease (claims), COPD and ischemic heart disease; pm2.5 and $\bar{\mathrm{pm}2.5}$ are the yearly and the mean values (calculated across the whole period) for PM_2.5_ concentrations at municipal level; β and γ denote the effects of time-varying and time-constant (e.g., average values) covariates respectively, and t represents the calendar year. Radon, Urbanization and Deprivation are categorical variables describing the constant values (or class of values) over the entire analyzed period of radon concentration, urbanization and deprivation levels as defined above. Consequently, the time-varying components were not included in the model. The dataset included the number of LC death counts and covariate values by year (2006–2015) and municipality (8092). The analysis was carried out considering males and females separately due to the potential differences in incidence in the two sets.

We considered both yearly and mean values for the adopted covariates/risk factors since, according to the longitudinal modelling literature (see e.g., [27]), two effects can be estimated in such a regression model. The first is a dynamic one (usually known in the demographic literature as the age effect), which can be associated to the (short-range) dynamics in the covariate/risk factor, defined as the departures of time-specific value of that variable from its mean value calculated over the period. The second one is essentially associated to the mean level calculated over the entire time period. It refers to the so called between or cohort effect, which represents a proxy of unit-specific unobserved risk factors associated to each observed source of variation. In that sense, mean values may be associated to long-range impact of potentially unobserved determinants. For mesothelioma, this is not a problem, as it is essentially associated with exposure to asbestos (even if different survival rates may somewhat bias the figure), while, for all the other covariates, caution is needed when interpreting the corresponding impact. A similar interpretation can also be considered for the mean PM_2.5_ values over the analyzed period, as this may indicate general exposure to pollutants and all municipality-specific features associated with it. We have fitted the negative binomial GLM for the LC death counts, using the corresponding expected counts as offset, considering both the mean level across the analyzed period and the yearly value as predictors, whenever possible (that is for all but radon, urbanization, and deprivation levels).

Association of LC and mesothelioma, occupational respiratory disease, PM_2.5_, and radon was assessed by the corresponding estimated effects in the fitted model, with the remaining covariates considered as adjustment factors. Increment risks (IR) of LC and confidence intervals were derived by regression coefficients using the following formula:

IR% = (exp (β × ∆) − 1) × 100

where ∆ represents the increment used for each predictor. We used a unit of increment for mesothelioma death and occupational respiratory disease rates per 1000 residents, and an increment of 10 µg/m^3^ for PM_2.5_. The number of expected LC mortality counts that can be attributed to each of the predictors was estimated by calculating the difference between the observed total LC death counts, and the LC death counts predicted by the model, setting to zero one predictor at a time. The predictors-attributed to the total LC deaths ratio was calculated as well (LC fraction). A counterfactual value of 5 µg/m^3^ was used for LC cases caused by PM_2.5_ exposure, as suggested elsewhere [28]. All analyses were performed using STATA 16 (StataCorp, College Station, TX, USA, 2019).

## 3. Results

### 3.1. Environmental Exposure

Table 2 reports some exploratory statistics concerning levels of concentrations for the environmental variables (PM_2.5_ and radon) considered in this study. Maps of mean concentrations are displayed in Supplementary Figures S1 and S2 of the supplementary materials (SM). They show a large spatial heterogeneity, particularly for PM_2.5_, with higher concentration levels in the Po valley region and in urban metropolitan areas. A mean PM_2.5_ value of 15.88 µg/m^3^ was observed across the country during the analyzed period, with a standard deviation of 6.35 µg/m^3^. The 75th and 95th percentiles are 20.9 and 27.8 µg/m^3^, respectively.

As for the radon exposure, a mean value of 80 Bq/m^3^, with a standard deviation of 58 Bq/m^3^, was estimated, with the 95th percentile equal to 181 Bq/m^3^. The number of municipalities for each of the identified radon class is also shown in Table 2. The time series of mean nationwide PM_2.5_ concentration is reported in Supplementary Figure S3 of the SM, where a decreasing trend can be observed since 2006.

### 3.2. Statistical Results and Model Estimates

Table 3 shows the observed crude LC mortality rate stratified by classes of observed predictors values. A total of 329,101 LC cases were identified from 2006 to 2015 (mortality rate: 55.4 per 100,000 persons), with higher rates for males rather than females (85.6 and 26.9 per 100,000 persons respectively). LC crude mortality rates increase with increasing rates of both mesothelioma deaths and occupational respiratory disease claims. Slowly increasing rates were associated to increasing COPD and ischemic heart disease death rates. As far as environmental covariates are entailed, LC crude mortality rates are slightly increasing with PM_2.5_ and present an almost flat trend with increasing of radon concentrations, urbanization, and deprivation levels; nonetheless, we have to consider that these are just marginal associations that do not take into account the joint impact of multiple potential risk factors.

Maps of mortality rates for LC and mesothelioma, as well as for claims related to occupational respiratory diseases are shown in Supplementary Figures S4–S6. Supplementary Figure S7 shows the time series of total lung cancer and mesothelioma death counts registered in Italy during 2006–2015. As for the LC mortality, after an increase from 2006 to 2010, we may observe a slightly decreasing trend. Conversely, mesothelioma death counts show a monotone (increasing) behaviour over the analyzed time window.

Supplementary Tables S2 and S3 show for males and females, respectively, the parameter estimates derived by the regression model defined above. After fitting this model, we realized that the most significant and stable effects were those associated to the mean levels, implying a long-term effect rather than a response to short-term changes (annual).

Therefore, the previous model equation was reduced to include only mean covariates values with the purpose of producing a more robust attributable fraction estimate. Model parameter estimates are reported in Supplementary Tables S4 and S5 for males and females, respectively. It should be noted that, due to orthogonality of yearly and mean value predictors, the results stay quite stable moving from the more complex to the simpler model specification.

### 3.3. Estimates for the Attributable Fraction

Based on the reduced model estimates reported in Supplementary Tables S4 and S5, we derived the estimated (adjusted) increment risk of lung cancer as (exp(b × ∆)−1) × 100 where b is the estimated coefficient and ∆ represents the increment used for each predictor as reported in Table 4. The corresponding, approximated, confidence intervals were also calculated. Whenever needed, these have been derived by applying a block bootstrap approach (with blocks identified by municipalities) to the observed data and deriving the corresponding estimates of number of expected cases for B = 1000 resamples. By comparing the observed LC death counts and the (model-based) predictions obtained by setting each predictor to a benchmark value, we obtained a simple, very crude, estimate of the LC death count fraction that can be attributed to each (current value of the) predictor.

**Figure 1:**
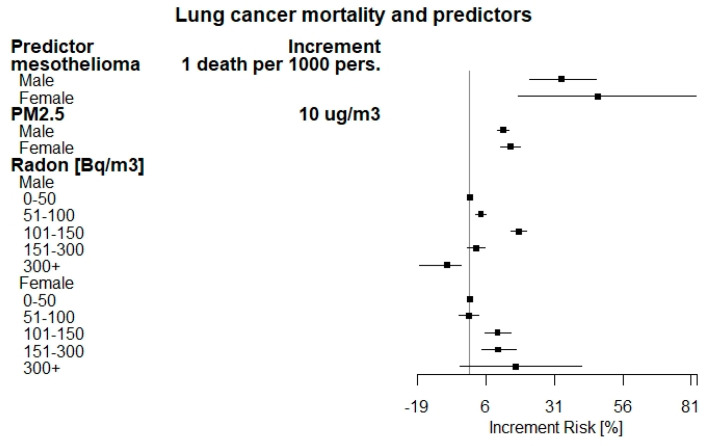
Forest plot of adjusted increment risk of lung cancer mortality by predictors.

Adjusted increment risks (IR) (%) of lung cancer by predictors in the period 2006–2015 are reported in Table 4. A forest plot of these estimates is shown in Figure 1. An increased risk was found by increment of one mesothelioma death per 1000 persons (33.59% (95% CI: 24.19, 43.71) for males, 46.74% (95% CI: 18.08, 82.36) for females).

A total of 2719 (95% CI: 2018–3419) and 436 (95% CI: 182, 689) LC cases have been estimated to be attributable to asbestos exposure (as proxied by mesothelioma death counts), with an estimated LC fraction of 1.10% (95% CI: 0.82–1.39) and 0.53% (95% CI: 0.22–0.83) for males and females, respectively. As for PM_2.5_ exposure, the increment risk for LC was found to be 12.33% (95% CI: 10.04, 14.33) and 14.96% (95% CI: 11.54, 18.49) for males and females, for an increment of 10 μ/m^3^ assuming a counterfactual value of 5 μg/m^3^. The LC fraction that has been estimated as attributable to PM_2.5_ exposure is 14.1% (95% CI: 12.47–15.68) and 16.2% (95% CI: 16.24–16.29) for males and females, accounting for about 47,996 LC mortality cases during the studied period. According to occupational respiratory diseases results, we observe that the estimate is not significant for both males and females and, therefore, no attributable fraction or IR can be estimated with satisfactory precision. The estimate obtained by considering all the data without stratifying by gender is not significant as well. The figures associated with the Radon concentration levels showed a marked increase at 101–150 Bq/m^3^ in males (17.98%) and over 300 Bq/m^3^ in females (16.75%) with respect to those exposed to concentrations up to 50 Bq/m^3^ assumed as reference level. Increment risks were observed also for the remaining classes of radon concentrations in both genders, except for the class over 300 Bq/m^3^ in males (which is however a very low frequency class), with a statistically significant decrease, and the class 51–100 Bq/m^3^ in females, with a not statistically significant effect. The LC fraction attributable to Radon exposure was 3.92% (95% CI: 3.49–4.20) and 1.5% (95% CI: 1.42–1.66) for males and females, accounting for 10,946 LC cases in total.

## 4. Discussion

Lung cancer is the most frequent malignant neoplasm in most countries, and the main cancer-related cause of mortality worldwide. According to the Global Burden of Disease Study (2016), during 2006–2016 lung cancer deaths increased from 1.44 to 1.71 million deaths, with a statistically significant annual percent change (APC) of 18.3 [29].

The geographic and temporal patterns of lung cancer incidence and mortality, at general population level, are largely determined by tobacco consumption, the principal etiological factor in lung carcinogenesis. Other factors such as genetic susceptibility, poor diet, occupational exposure, and air pollution may act, independently or jointly with tobacco smoking in influencing the epidemiology of lung cancer. To support the efficiency of the welfare and insurance systems, the evaluation of predictors for lung cancer mortality, in particular those with an occupational and environmental nature, results in great relevance.

The present study consists in an ecological analysis carried out at municipal level in Italy, with the aim of evaluating the contribution of selected predictive factors for lung cancer mortality. The mortality for chronic obstructive pulmonary disease (COPD) and ischemic heart diseases were considered as proxies of smoking habits at territorial level, according to the positive association observed for both diseases [30]. COPD is the most common smoking-related disease and the presence of COPD increases the incidence of lung cancer [31]. However, some studies have shown that the effect of COPD for lung cancer development is independent of smoking exposure, with a four to sixfold greater risk of developing lung cancer when compared to matched smokers with normal lung function [32].

The lung is the target organ for several substances and compounds that are recognized as human carcinogens, especially in occupational settings. The largest attributable fractions were estimated in highly industrialized areas with a great prevalence of shipbuilding and railroad equipment manufacturing, metal basic and chemical industries [33]. In the UK and France, proportions of lung cancer cases that can be attributed to occupational agents, were estimated at 14.5% [34] and 12.5% (in men) respectively [35]. However, there is still uncertainty about the distribution of exposure across occupations, and confounding by smoking may have a major role.

In this study, the compensated cases of occupational respiratory diseases (ICD10: J40-J47 and J60-J67) have been included in the model as a measure of occupational exposure to lung carcinogen agents at municipal level. The increasing lung cancer mortality rates seem to be associated with increasing number of compensated cases (see Table 3), but when used as predictor, the corresponding estimates are not significant, likely due to the great variability in the procedures for compensation. Therefore, we were not able to estimate a reliable occupational attributable fraction of lung cancer deaths. This point deserves future research, as occupational carcinogens are of great interest.

Lung cancer mortality is strongly associated with asbestos exposure as reported in epidemiological and ecological studies in several countries including Great Britain [6], Italy [10], Argentina, Brazil, Colombia, and Mexico [36]. Currently, asbestos production worldwide is about 2 million tons and it is still widely used in developed and emerging economy countries [37]. The use of asbestos in Italy ceased following a law-compulsory ban on production, import, export, use, and trading in 1992. The present study, as well as a previous ecological study carried out in Piemonte region [9] was based on municipalities and used mesothelioma mortality as a proxy of asbestos exposure to estimate the fraction of lung cancer deaths that can be attributed to asbestos. This mechanism is of great importance and it is not likely suffering from confounding, as mesothelioma is essentially motivated by exposure to asbestos. The fraction of lung cancer that is estimated to be attributable to asbestos exposure is 1.1% (95% CI: 0.82–1.39) and 0.53% (95% CI: 0.22–0.83) for males and females, respectively. Similar results were found in a previous Italian ecological study, where the fraction of all lung cancer deaths in males that can be attributed to asbestos was estimated to be 1.6–3.7% (between 380 and 770 deaths per year) [10]. However, the results by gender should be handled with care. While gender is known to be a factor influencing mesothelioma survival rates (see e.g., [38]), which may suggest that related death counts may reflect a differential exposure to asbestos, we need to point out that mesothelioma counts are quite low for women and, therefore, the corresponding impact estimate may not be considered as robust as that for men (as suggested by the wider confidence interval). In addition, the results have to be evaluated considering the different latency period between asbestos exposure and mesothelioma or lung cancer occurrence [39,40,41].

In the present study, the highest mortality rates were observed in intermediate categories of deprivation (medium and deprived), similarly to ecological studies in Sweden [42] and Germany [43]. The present study found increasing IR estimates with increasing deprivation levels, at least for males (Supplementary Table S1).

The present study used urbanization as another important predictor of lung cancer. This is a multifactorial indicator that considers sociodemographic and urban characteristics. In this study crude lung cancer mortality rates did not present clear differences among clusters of urbanization, except for the highest (but also less frequent) one, with greater mortality rate. However, the model estimates higher risks for mostly urbanized areas for both males and females (see Supplementary Table S1). A greater density of resident population and multiple emission sources, driving poor ambient air pollution, can motivate the unhealthy effects related to urban environment.

Air pollution is a complex mixture of different gaseous and particulate components: particulate matter (PM) and NO_2_ are two well-characterized air pollutants. The transport and effects of particulate matter, both in the atmosphere and in the human respiratory tract, are ruled principally by particulate size, shape, and toxicity. Data indicate that fine particulate matter is strongly associated with acute and chronic adverse health outcomes. Our findings suggest that PM_2.5_ exposure is associated with an increase in the risk of LC mortality (12.10–14.96% for an increment of 10 µg/m^3^). The fraction of LC cases that is estimated to be attributable to PM_2.5_ is 14.05% (95% CI: 12.47, 15.68) and 16.26% (95% CI: 16.24, 16.29) for males and females, respectively. A recent systematic review and meta-analysis found a relative risk ratio of 1.12 of lung cancer mortality per increment of 10 µg/m^3^ of PM_2.5_ [18] close to the values estimated in the current study. Similar results were also found in a Chinese study, which analyzed the association of fine particulate matter and lung cancer mortality [23]. In a critical review among 17 articles about LC mortality and particulate matter, Wang et al. [17] found relative risks ranging from 1.08 to 1.60 per increments of 10 µg/m^3^ of PM_2.5_. Former systematic review and meta-analysis quantified the relative risk to 1.09 [44]. The findings of the present study are consistent with the above results.

Another leading environmental cause of lung cancer mortality is the prolonged exposure to radon. Substantial evidence of radon carcinogenicity came from 11 large epidemiologic studies of underground miners exposed to radon and from a pooled analysis of those studies [45,46]. Further pooled analyses of residential radon studies performed in China, Europe, and North America have also demonstrated that prolonged exposure to radon (and radon decay products), even below the EPA’s action level, may significantly increase lung cancer risk. The percentages of lung cancer deaths attributable to radon exposure found in this study were 3.9% in males and 1.6% in females. However, caution is needed as these estimates are based on very sparse data and, therefore, they may be local. Such results are in line with those provided by the literature [12,47]. When considering radon risk, we must consider that ecologic studies are affected by limitations deriving from inaccurate measurement of actual exposure as well as to the confounding presence of smoking habits. The strong synergism between radon exposure and smoking as risk factors is in fact a critical aspect of the relationship between radon and lung cancer [48].

We analyzed the associations of predictors with LC mortality by separately processing the two genders due to the differences in incidence rates. According to the different crude mortality rates (86 and 27 for male and female respectively), we cannot exclude possible different bias or lack of sufficient information for women, as it can be evinced from the larger confidence intervals. As far as the results by gender are concerned, we found some differences in IR between males and females. We found slightly higher risks in women than men, although the contributions to the total number of LC deaths are opposite due to the higher crude mortality rate in men. Tobacco use and lifestyles may conceivably be confounding factors in these associations. Environmental aspects like ambient asbestos, PM_2.5_, and radon exposures may have a role in such differences as well. Differences in relative risks between genders were also estimated in studies about the association of lung cancer with radon [24] and air pollution [49].

This study has several strengths and limitations. First, we adopt a comprehensive national ecological study by trying to include the most relevant factors, or their proxies, involved in LC mortality. Among them, we should mention the availability of long-term (2006–2015) data of PM_2.5_ exposure over the whole national territory. Because of the long latency from exposure to lung cancer incidence and mortality, long-term data on PM exposure are mandatory. In addition, we have included, for the first time, data on claims for occupational respiratory diseases registered at national level by the national workers’ compensation authority. This allows considering the occupational component occurring in lung cancer mortality, even if such claim data may not equally cover men and women due to the specific features of the Italian job market (unregistered jobs, differential participation of women to the job market, etc.). This point, however, is a first approach to try to estimate the fraction of total LC cases due to occupational exposure, and it deserves further critical analysis.

As for limitations, we should consider that we have not directly included important determinants of lung cancer mortality like smoking habits, diet, obesity, and lifestyles. The inclusion of such data at individual bases is impossible for an ecological study like this one, based on mortality and compensation registries. Another important limitation is the heterogeneity of radon data and the lack for long-term variation. Obtaining long-term radon exposure data at municipal level for the entire national territory is almost unfeasible. Radon exposure data are collected at different locations (residential, school, workplace) and times using different methods (measurements, estimations, and geo-spatial analysis). By integrating all available information, we have built a measure of radon exposure at municipal levels; however, considering the different materials and methods used, a large heterogeneity and poor accuracy is expected. We tried to limit the impact by defining classes of radon concentrations. Last, we should remark that the estimated effects are associated to the mean values of predictors over the whole analyzed period. Therefore, they should not be considered as causal effects but, rather, as effects associated to these predictors or, better, to potentially unobserved characteristics of municipalities associated with these predictors.

## 5. Conclusions

Lung cancer etiology is complex, multifactorial, and involves occupational, environmental determinants as well as life habits. The correct estimation of the role of the different factor is a substantial tool for defining the prevention policies and for supporting the occupational insurance and welfare system. In Italy, the recent availability of pollution exposure data at high spatial resolution, has suggested to define a multivariate statistical model for estimating the lung cancer mortality by predictors in an ecological design context. The findings confirm the substantial role of both environmental and asbestos related factors in lung cancer risk and to some extent the occupational ones. The residential exposure to radon, PM_2.5_ pollution, and the other occupational lung carcinogens agents are still key factors of risk for lung cancer mortality. The identification of environmental and occupational dimensions of lung cancer risk is crucial for improving awareness of the origin of the disease, leading to increase the equity of welfare system.

## Figures and Tables

**Table 1:** List of variables used in the study (ISTAT: Italian National Institute of Statistics; INAIL: Italian Workers’ Compensation Authority; COPD: Chronic Obstructive Pulmonary Disease; BEEP: Big data in Environmental and occupational Epidemiology).

Variable	Description	Source	Temporal Resolution	Spatial Resolution
Lung cancer mortality	Malignant neoplasm trachea, bronchus, and lung (ICD-10 codes: C33-C34)	ISTAT	daily	Municipality
Mesothelioma mortality	Malignant mesothelioma (ICD-10 codes: C45)	ISTAT	daily	Municipality
Ischemic heart mortality	Ischemic heart diseases (ICD-10 codes: I20-I25)	ISTAT	daily	Municipality
COPD mortality	Chronic obstructive pulmonary disease and other respiratory conditions (ICD-10 codes: J40-J44)	ISTAT	daily	Municipality
Occupational respiratory diseases	Compensated cases for occupational respiratory diseases (ICD-10 codes: J40-J47, J60-J67)	INAIL	daily	Municipality
PM_2.5_	Concentration of PM_2.5_	BEEP project	daily	1 × 1 km
Radon	Concentration of Radon	Local Environmental Authorities data and [20]	constant	Municipality
Urbanization level	Index of urbanization levels	[21]	constant	Municipality
Deprivation level	Index of deprivation levels	[22]	constant	Municipality
Population	Amount of population	ISTAT	Annual	Municipality

**Table 2:** Environmental variables: exploratory statistics, 2006–2015 data.

	Min	Max		Mean	SD	Percentiles						
						5th	25th		50th	75th	95th	
PM_2.5_ (µg/m^3^)	5.85	36.28		15.88	6.35	8.20	10.75		13.92	20.90	27.83	
Radon (Bq/m^3^)	9	1008		80.33	58.59	18	44		69	102	181
Radon levels (Bq/m^3^)			Municipalities									
			N.					%				
0–50			2569					31.7				
51–100			3459					42.7				
101–150			1455					18.0				
151–300			537					6.6				
>300			72					0.9				

**Table 3:** Crude lung cancer mortality counts/rates by predictor strata.

Predictor		Lung Cancer
	Cases	Crude Mortality RATE (per 100,000)
Total	329,101	55.4
Gender		
Male	246,555	85.6
Female	82,546	26.9
Mesothelioma (death counts)		
0	192,421	47.0
1–20	117,170	66.4
21–40	16,209	93.8
41–60	2623	118.9
>60	678	121.9
PM_2.5_ (µg/m^3^)		
5–10	15,536	47.0
11–15	109,296	51.0
15–20	85,884	60.2
20–25	58,487	57.0
>25	59,898	58.9
COPD (death counts)		
0	51,551	45.3
1–5	121,109	51.6
6–10	37,113	58.1
10–15	18,701	64.5
>15	100,627	68.6
Ischemic heart disease (death counts)		
0	14,689	50.8
1–20	168,570	51.3
21–40	35,791	56.5
41–60	16,537	60.8
>60	93,514	66.9
Occupational Resp. disease (claims)		
0	269,673	51.6
1–2	40,362	78.1
3–4	10,265	87.8
5–6	1504	105.0
>6	7297	100.6
Radon (Bq/m^3^)		
0–50	139,633	54.2
51–100	126,914	56.9
101–150	49,264	55.4
151–300	11,950	55.2
>300	1340	43.0
Urbanization level		
low	13,622	51.2
low-medium	21,848	50.4
medium	35,891	51.1
medium-high	61,165	51.8
high	196,575	58.4
Deprivation level		
very rich	64,321	52.4
rich	64,321	52.4
medium	71,489	60.9
deprived	67,997	58.0
very deprived	55,494	48.5

**Table 4:** Estimates of adjusted * Increment Risk (%) (approximate 95% CI) of lung cancer (LC) mortality, expected LC cases and attributable LC fraction by predictor, 2006–2015.

Predictor	Incr	Unit	IR% (95% CI)	Exp. LC Cases(95% CI)	LC Fraction (%) (95% CI)
Mesothelioma					
Male	1	Death per 1000 pers.	33.59 (21.98, 46.30)	2719 (2018, 3419)	1.10 (0.82, 1.39)
Female			46.74 (17.86, 82.70)	436 (182, 689)	0.53 (0.22, 0.83)
PM_2.5_					
Male	10	µg/m^3^	12.33 (10.04, 14.33)	34,641 (30,738, 38,657)	14.05 (12.47, 15.68)
Female			14.96 (11.54, 18.49)	13,424 (13,402, 13,447)	16.26 (16.24, 16.29)
Occupational respiratory disease					
Male	1	Disease per 1000 pers.	ns	n.a.	n.a.
Female			ns	n.a.	n.a.
Radon (Bq/m^3^)					
Male				9669 (8605, 10,346)	3.92 (3.49, 4.20)
0–50			0.00		
51–100			4.12 (2.38, 5.89)		
101–150			17.98 (15.26, 20.76)		
151–300			2.44 (−0.78, 5.77)		
300+			−8.20 (−18.17, −2.99)		
Female				1277 (1174, 1368)	1.56 (1.42, 1.66)
0–50			0.00		
51–100			−0.27 (−3.72, 3.31)		
101–150			10.23 (5.52, 15.15)		
151–300			10.50 (4.44, 16.90)		
300+			16.76 (−3.30, 40.98)		

* Adjusted for deprivation and urbanization levels, and mortality from COPD and ischemic heart disease.

## Supplementary Materials

The following are available online at https://www.mdpi.com/1660-4601/18/4/1896/s1, Figure S1: Mean concentration of PM2.5, Figure S2: Mean concentration of Radon, Figure S3: Time series of yearly mean nationwide PM2.5 concentration, Figure S4: LC mortality rate per 100 k persons by Province, Figure S5: Mesothelioma mortality rate per 100 k persons by Province, Figure S6: Occupational respiratory disease rate per 100k persons by Province, Figure S7: Time series of total lung cancer cases and mesothelioma cases occurring at national level during years 2006–2015, Table S1: Increment Risk [%] (95% CI) of lung cancer (LC) mortality by Deprivation and Urbanization. 2006–2015, Table S2: Full model for LC death counts. Males, years 2006–2015, parameter estimates, Table S3: Full model for LC death counts. Females, years 2006–2015, parameter estimates, Table S4: Reduced model for LC death counts. Males, years 2006–2015, parameter estimates, Table S5: Reduced model for LC death counts. Females, years 2006–2015, parameter estimates.
