# Supplementary material for: Predictors of Lung Cancer Risk: An Ecological Study Using Mortality and Environmental Data by Municipalities in Italy
Source: Int J Environ Res Public Health. 2021 Feb 16;18(4):1896. doi: 10.3390/ijerph18041896 (PMC7922734; doi:10.3390/ijerph18041896)
Supplement: Supplementary file 1 [file ijerph-18-01896-s001.docx]

**Supplementary Material of paper entitled:**

**Predictors of lung cancer risk: an ecological study using mortality and environmental data by municipalities in Italy**


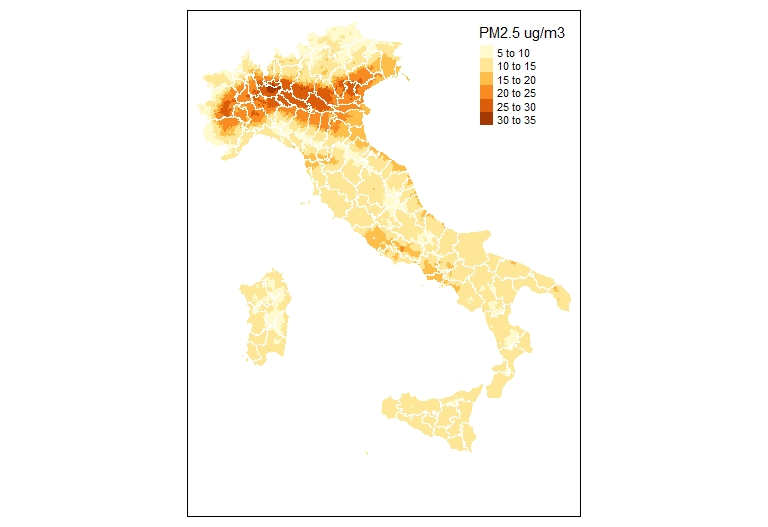


Figure S1. Mean concentration of PM_2.5_. Provinces borders are shown as white lines. 2006-2015 data.


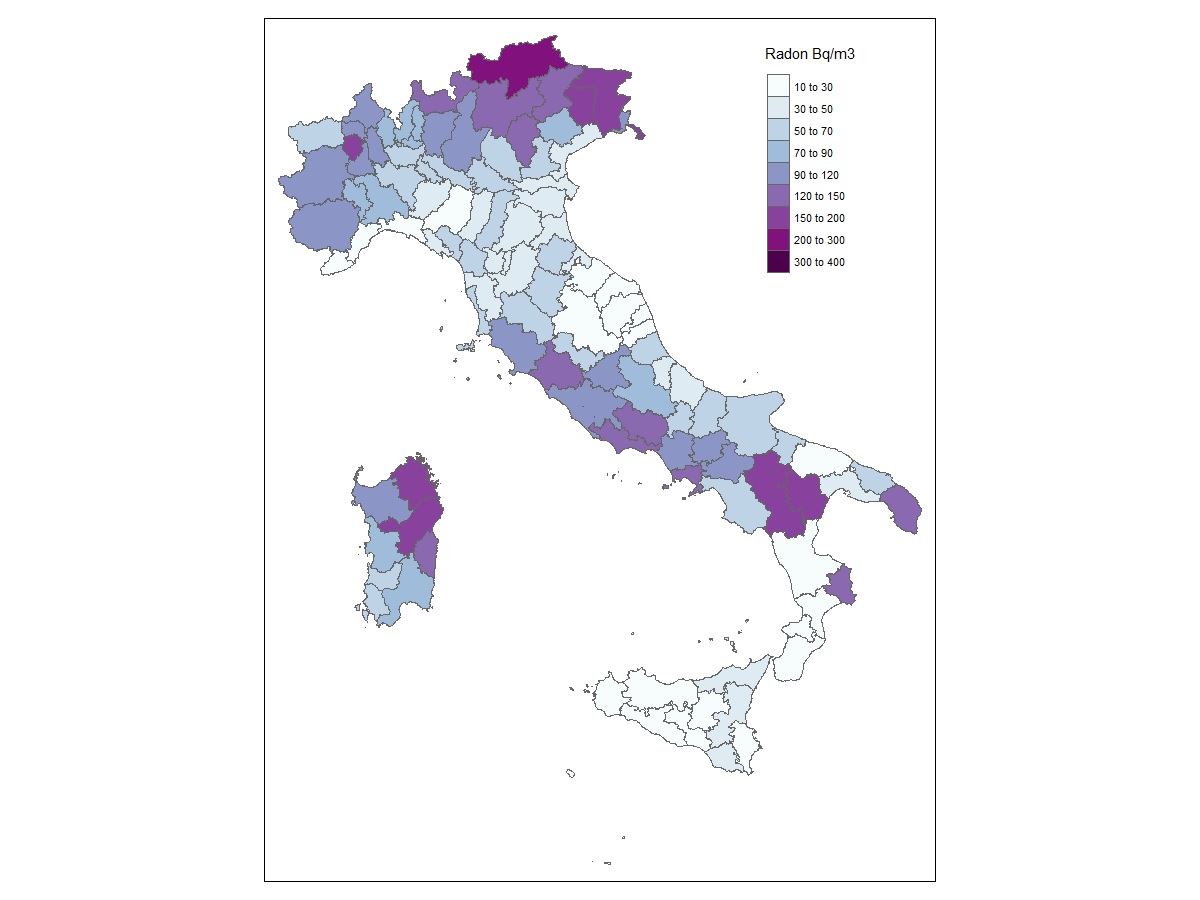


Figure S2. Mean concentration of Radon. Provinces borders are shown as black lines.


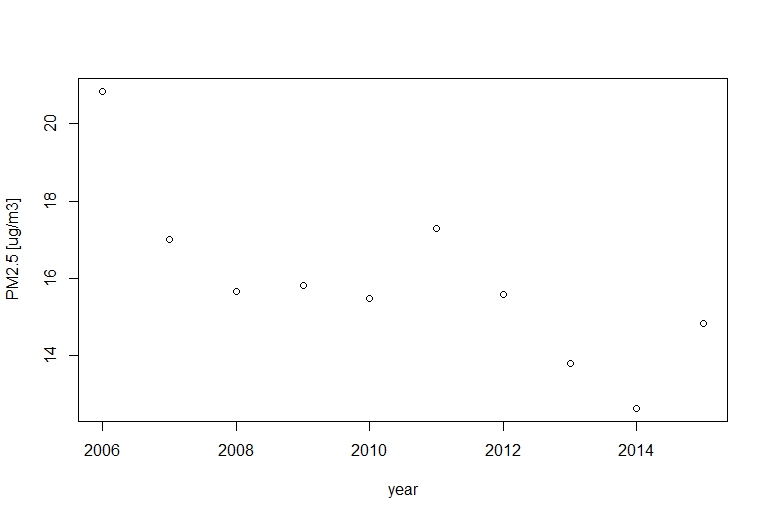


Figure S3. Time series of yearly mean nationwide PM_2.5_ concentration.


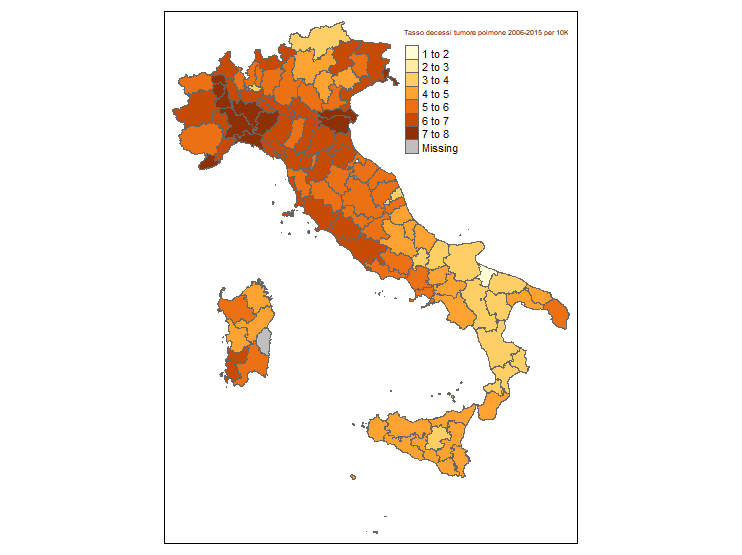


Figure S4. LC mortality rate per 100k persons by Province. 2006-2015 data.


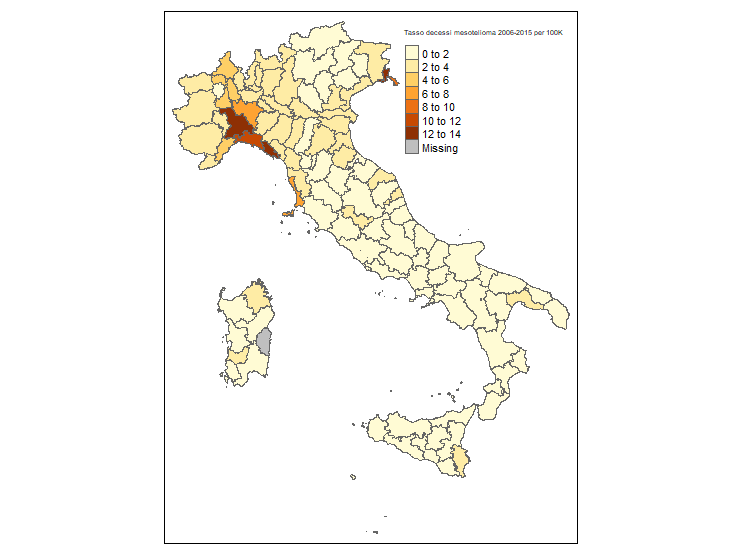


Figure S5. Mesothelioma mortality rate per 100k persons by Province. 2006-2015 data.


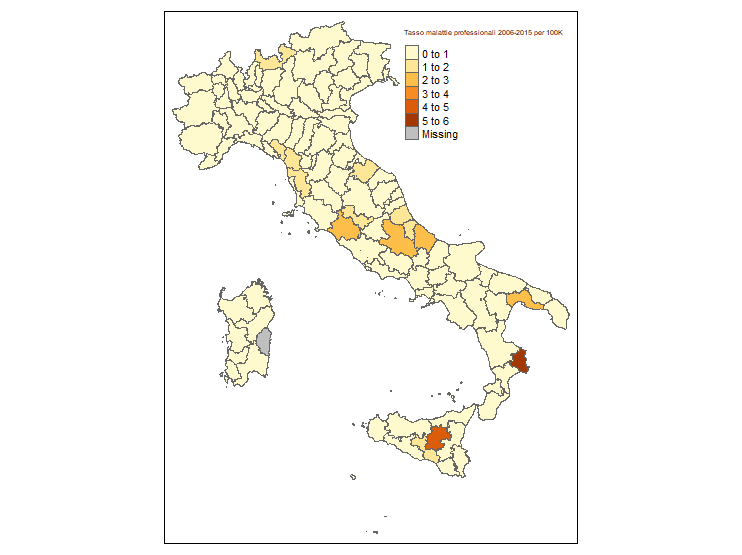


Figure S6. Occupational respiratory disease rate per 100k persons by Province. 2006-2015 data.

| 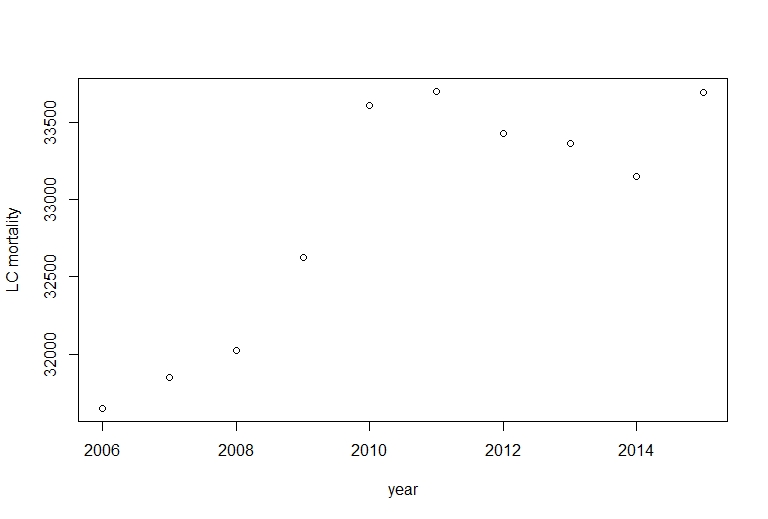 |
| --- |
| 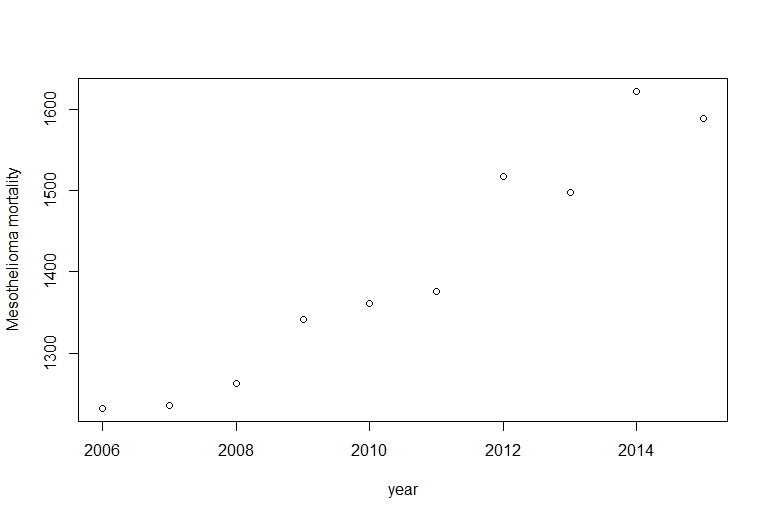 |

Figure S7. Time series of total lung cancer cases (top) and mesothelioma cases (bottom) occurring at national level during years 2006-2015.

Table S1. Increment Risk [%] (95% CI) of lung cancer (LC) mortality by Deprivation and Urbanization. 2006-2015.

| **Predictor** | **Beta** | **STD** | **IR% (95% CI)** |
| --- | --- | --- | --- |
| Deprivation |  |  |  |
| *Male* |  |  |  |
| very rich |  |  | 0 |
| rich | 0.0479 | 0.0095 | 4.90 (2.97, 6.87) |
| medium | 0.0793 | 0.0104 | 8.25 (6.07, 10.48) |
| deprived | 0.0889 | 0.0118 | 9.30 (6.80, 11.86) |
| very deprived | 0.1708 | 0.0135 | 18.62 (15.52, 21.80) |
| *Female* |  |  |  |
| very rich |  |  | 0 |
| rich | 0.0177 | 0.0165 | 1.78 (-1.46, 5.13) ns |
| medium | -0.0153 | 0.0185 | -1.52 (-5.02, 2.11) ns |
| deprived | -0.0946 | 0.0256 | -9.03 (-13.48, -4.35) |
| very deprived | -0.1901 | 0.0271 | -17,31 (-21.58, -12.81) |
| Urbanization |  |  |  |
| *Male* |  |  |  |
| low |  |  | 0 |
| low-medium | 0.0301 | 0.0177 | 3.06 (-0.45, 6.69) ns |
| medium | 0.0878 | 0.0168 | 9.17 (5.63, 12.83) |
| medium-high | 0.1041 | 0.0169 | 10.97 (7.36, 14.70) |
| high | 0.1142 | 0.0179 | 12.10 (8.24, 16.09) |
| *Female* |  |  |  |
| low |  |  | 0 |
| low-medium | 0.0232 | 0.0302 | 2.35 (-3.54, 8.59) ns |
| medium | 0.1217 | 0.0301 | 12.94 (6.47, 19.81) |
| medium-high | 0.1676 | 0.0285 | 18.25 (11.83, 25.03) |
| high | 0.3034 | 0.0311 | 35.45 (27.45, 43.95) |

- ns: statistically not significant estimate

Table S2. Full model for LC death counts. Males, years 2006-2015, parameter estimates. In bold the statistically significant parameters.

| **Variable** | **Coef** | **SE(robust)** | **z** | **P(>\|z\|)** | **Lower Bound (95% CI)** | **Upper Bound (95% CI)** |
| --- | --- | --- | --- | --- | --- | --- |
| $Year$ |  |  |  |  |  |  |
| 2007 | -0.0031 | 0.0113 | -0.27 | 0.784 | -0.0252 | 0.0190 |
| 2008 | -0.0094 | 0.0118 | -0.8 | 0.426 | -0.0325 | 0.0137 |
| 2009 | 0.0007 | 0.0115 | 0.06 | 0.95 | -0.0217 | 0.0232 |
| 2010 | **-0.0294** | **0.0120** | **-2.46** | **0.014** | **-0.0529** | **-0.0060** |
| 2011 | **-0.0263** | **0.0104** | **-2.53** | **0.011** | **-0.0466** | **-0.0059** |
| 2012 | **-0.0280** | **0.0118** | **-2.37** | **0.018** | **-0.0511** | **-0.0048** |
| 2013 | **-0.0308** | **0.0128** | **-2.41** | **0.016** | **-0.0558** | **-0.0058** |
| 2014 | **-0.0299** | **0.0145** | **-2.06** | **0.039** | **-0.0584** | **-0.0015** |
| 2015 | **-0.0253** | **0.0120** | **-2.1** | **0.035** | **-0.0488** | **-0.0017** |
|  |  |  |  |  |  |  |
| $TOccupResp$ | 0.0015 | 0.0323 | 0.05 | 0.962 | -0.0619 | 0.0649 |
| $\overline{TOccupResp}$ | 0.0997 | 0.0982 | 1.02 | 0.31 | -0.0928 | 0.2922 |
| $TMesothelioma$ | -0.0195 | 0.0191 | -1.02 | 0.307 | -0.0569 | 0.0179 |
| $\overline{TMesothelioma}$ | **0.3088** | **0.0498** | **6.2** | **0** | **0.2113** | **0.4064** |
| $TIschemHeart$ | 0.0004 | 0.0032 | 0.11 | 0.91 | -0.0058 | 0.0065 |
| $\overline{TischemHeart}$ | **0.0225** | **0.0078** | **2.89** | **0.004** | **0.0072** | **0.0378** |
| $TCopd$ | -0.0102 | 0.0052 | -1.95 | 0.051 | -0.0204 | 0.0000 |
| $\overline{TCopd}$ | 0.0189 | 0.0146 | 1.29 | 0.196 | -0.0098 | 0.0476 |
| ${pm}_{2.5}$ | -0.0013 | 0.0012 | -1.06 | 0.291 | -0.0038 | 0.0011 |
| $\overline{{pm}_{2.5}}$ | **0.0129** | **0.0015** | **8.39** | **0** | **0.0099** | **0.0160** |
|  |  |  |  |  |  |  |
| $Urbanization$ |  |  |  |  |  |  |
| 2 | 0.0301 | 0.0177 | 1.7 | 0.089 | -0.0045 | 0.0647 |
| 3 | **0.0878** | **0.0168** | **5.22** | **0** | **0.0548** | **0.1207** |
| 4 | **0.1040** | **0.0169** | **6.16** | **0** | **0.0710** | **0.1371** |
| 5 | **0.1142** | **0.0179** | **6.39** | **0** | **0.0792** | **0.1492** |
|  |  |  |  |  |  |  |
| $Deprivation$ |  |  |  |  |  |  |
| 2 | **0.0479** | **0.0095** | **5.05** | **0** | **0.0293** | **0.0665** |
| 3 | **0.0793** | **0.0104** | **7.62** | **0** | **0.0589** | **0.0997** |
| 4 | **0.0889** | **0.0118** | **7.53** | **0** | **0.0658** | **0.1121** |
| 5 | **0.1708** | **0.0135** | **12.65** | **0** | **0.1443** | **0.1973** |
|  |  |  |  |  |  |  |
| $Radon$ |  |  |  |  |  |  |
| [50,100) | **0.0404** | **0.0086** | **4.69** | **0** | **0.0235** | **0.0573** |
| [100,150) | **0.1653** | **0.0119** | **13.9** | **0** | **0.1420** | **0.1886** |
| [150,300) | 0.0241 | 0.0163 | 1.48 | 0.14 | -0.0079 | 0.0561 |
| [300+) | -0.0856 | 0.0587 | -1.46 | 0.145 | -0.2006 | 0.0294 |

Table S3. Full model for LC death counts. Females, years 2006-2015, parameter estimates. In bold the statistically significant parameters.

| **Variable** | **Coef** | **SE(robust)** | **z** | **P(>\|z\|)** | **Lower Bound (95% CI)** | **Upper Bound (95% CI)** |
| --- | --- | --- | --- | --- | --- | --- |
| $Year$ |  |  |  |  |  |  |
| 2007 | -0.0225 | 0.0209 | -1.08 | 0.2800 | -0.0635 | 0.0184 |
| 2008 | -0.0177 | 0.0216 | -0.82 | 0.4140 | -0.0601 | 0.0247 |
| 2009 | -0.0209 | 0.0210 | -0.99 | 0.3200 | -0.0621 | 0.0203 |
| 2010 | -0.0335 | 0.0217 | -1.55 | 0.1220 | -0.0759 | 0.0090 |
| 2011 | -0.0269 | 0.0190 | -1.41 | 0.1570 | -0.0641 | 0.0104 |
| 2012 | -0.0334 | 0.0211 | -1.58 | 0.1140 | -0.0749 | 0.0080 |
| 2013 | -0.0213 | 0.0231 | -0.92 | 0.3570 | -0.0666 | 0.0240 |
| 2014 | -0.0309 | 0.0260 | -1.18 | 0.2360 | -0.0819 | 0.0202 |
| 2015 | -0.0269 | 0.0214 | -1.26 | 0.2080 | -0.0689 | 0.0150 |
|  |  |  |  |  |  |  |
| $TOccupResp$ | 0.2395 | 0.1798 | 1.33 | 0.1830 | -0.1129 | 0.5920 |
| $\overline{TOccupResp}$ | -1.3478 | 0.6946 | -1.94 | 0.0520 | -2.7091 | 0.0135 |
| $TMesothelioma$ | -0.0881 | 0.0604 | -1.46 | 0.1450 | -0.2064 | 0.0303 |
| $\overline{TMesothelioma}$ | **0.4669** | **0.1224** | **3.81** | **0** | **0.2269** | **0.7068** |
| $TIschemHeart$ | -0.0004 | 0.0055 | -0.07 | 0.9480 | -0.0112 | 0.0104 |
| $\overline{TischemHeart}$ | **0.0536** | **0.0119** | **4.49** | **0.0000** | **0.0302** | **0.0770** |
| $TCopd$ | 0.0062 | 0.0108 | 0.58 | 0.5640 | -0.0149 | 0.0274 |
| $\overline{TCopd}$ | **0.2203** | **0.0317** | **6.96** | **0.0000** | **0.1582** | **0.2823** |
| ${pm}_{2.5}$ | -0.0015 | 0.0023 | -0.65 | 0.5150 | -0.0059 | 0.0030 |
| $\overline{{pm}_{2.5}}$ | **0.0154** | **0.0027** | **5.65** | **0.0000** | **0.0101** | **0.0208** |
|  |  |  |  |  |  |  |
| $Urbanization$ |  |  |  |  |  |  |
| 2 | 0.0232 | 0.0302 | 0.77 | 0.4420 | -0.0360 | 0.0824 |
| 3 | **0.1217** | **0.0301** | **4.04** | **0.0000** | **0.0627** | **0.1807** |
| 4 | **0.1676** | **0.0285** | **5.89** | **0.0000** | **0.1118** | **0.2234** |
| 5 | **0.3035** | **0.0311** | **9.77** | **0.0000** | **0.2426** | **0.3644** |
|  |  |  |  |  |  |  |
| $Deprivation$ |  |  |  |  |  |  |
| 2 | 0.0177 | 0.0165 | 1.07 | 0.2840 | -0.0147 | 0.0501 |
| 3 | -0.0154 | 0.0185 | -0.83 | 0.4050 | -0.0516 | 0.0208 |
| 4 | **-0.0947** | **0.0256** | **-3.7** | **0.0000** | **-0.1448** | **-0.0445** |
| 5 | **-0.1901** | **0.0271** | **-7.02** | **0.0000** | **-0.2432** | **-0.1371** |
|  |  |  |  |  |  |  |
| $Radon$ |  |  |  |  |  |  |
| [50,100) | -0.0027 | 0.0180 | -0.15 | 0.88 | -0.0380 | 0.0325 |
| [100,150) | **0.0973** | **0.0223** | **4.37** | **0.0000** | **0.0537** | **0.1410** |
| [150,300) | **0.0998** | **0.0288** | **3.47** | **0.0010** | **0.0434** | **0.1562** |
| [300+) | 0.1549 | 0.0962 | 1.61 | 0.1070 | -0.0336 | 0.3435 |

Table S4. Reduced model for LC death counts. Males, years 2006-2015, parameter estimates. In bold the statistically significant parameters.

| **Variabile** | **Coef** | **SE(robust)** | **z** | **P(>\|z\|)** | **Lower Bound (95% CI)** | **Upper Bound (95% CI)** |
| --- | --- | --- | --- | --- | --- | --- |
| $Year$ |  |  |  |  |  |  |
| *2007* | 0.0022 | 0.0099 | 0.22 | 0.827 | -0.0172 | 0.0215 |
| 2008 | -0.0028 | 0.0098 | -0.28 | 0.778 | -0.0219 | 0.0164 |
| 2009 | 0.0064 | 0.0099 | 0.65 | 0.514 | -0.0129 | 0.0257 |
| 2010 | **-0.0229** | **0.0099** | **-2.32** | **0.02** | **-0.0423** | **-0.0036** |
| 2011 | **-0.0226** | **0.0097** | **-2.33** | **0.02** | **-0.0415** | **-0.0036** |
| 2012 | **-0.0215** | **0.0098** | **-2.2** | **0.028** | **-0.0408** | **-0.0023** |
| 2013 | **-0.0221** | **0.0098** | **-2.26** | **0.024** | **-0.0414** | **-0.0029** |
| 2014 | **-0.0193** | **0.0100** | **-1.92** | **0.055** | **-0.0389** | **0.0004** |
| 2015 | **-0.0186** | **0.0099** | **-1.87** | **0.062** | **-0.0381** | **0.0009** |
|  |  |  |  |  |  |  |
| $\overline{TMesothelioma}$ | **0.2896** | **0.0464** | **6.25** | **0** | **0.1987** | **0.3805** |
| $\overline{TOccupResp}$ | 0.1011 | 0.0946 | 1.07 | 0.285 | -0.0843 | 0.2865 |
| $\overline{TCopd}$ | 0.0089 | 0.0138 | 0.65 | 0.516 | -0.0181 | 0.0359 |
| $\overline{TischemHeart}$ | **0.0229** | **0.0072** | **3.17** | **0.002** | **0.0087** | **0.0370** |
| $\overline{{pm}_{2.5}}$ | **0.0116** | **0.0009** | **12.89** | **0** | **0.0099** | **0.0134** |
|  |  |  |  |  |  |  |
| $Urbanization$ |  |  |  |  |  |  |
| 2 | 0.0301 | 0.0177 | 1.7 | 0.089 | -0.0045 | 0.0648 |
| 3 | **0.0878** | **0.0168** | **5.22** | **0** | **0.0548** | **0.1207** |
| 4 | **0.1041** | **0.0169** | **6.16** | **0** | **0.0710** | **0.1371** |
| 5 | **0.1142** | **0.0179** | **6.39** | **0** | **0.0792** | **0.1492** |
|  |  |  |  |  |  |  |
| $Deprivation$ |  |  |  |  |  |  |
| 2 | **0.0479** | **0.0095** | **5.04** | **0** | **0.0293** | **0.0665** |
| 3 | **0.0793** | **0.0104** | **7.62** | **0** | **0.0589** | **0.0997** |
| 4 | **0.0889** | **0.0118** | **7.53** | **0** | **0.0658** | **0.1121** |
| 5 | **0.1708** | **0.0135** | **12.65** | **0** | **0.1443** | **0.1972** |
|  |  |  |  |  |  |  |
| $Radon$ |  |  |  |  |  |  |
| [50,100) | **0.0404** | **0.0086** | **4.68** | **0** | **0.0235** | **0.0573** |
| [100,150) | **0.1653** | **0.0119** | **13.9** | **0** | **0.1420** | **0.1887** |
| [150,300) | 0.0241 | 0.0163 | 1.48 | 0.139 | -0.0079 | 0.0561 |
| [300+) | -0.0856 | 0.0587 | -1.46 | 0.145 | -0.2005 | 0.0294 |

Table S5. Reduced model for LC death counts. Females, years 2006-2015, parameter estimates. In bold the statistically significant parameters.

| **Variable** | **Coef** | **SE(robust)** | **z** | **P(>\|z\|)** | **Lower Bound (95% CI)** | **Upper Bound (95% CI)** |
| --- | --- | --- | --- | --- | --- | --- |
| $Year$ |  |  |  |  |  |  |
| 2007 | -0.0170 | 0.0187 | -0.91 | 0.363 | -0.0537 | 0.0197 |
| 2008 | -0.0103 | 0.0184 | -0.56 | 0.576 | -0.0462 | 0.0257 |
| 2009 | -0.0141 | 0.0184 | -0.76 | 0.445 | -0.0501 | 0.0220 |
| 2010 | -0.0260 | 0.0182 | -1.43 | 0.153 | -0.0617 | 0.0097 |
| 2011 | -0.0229 | 0.0179 | -1.28 | 0.202 | -0.0580 | 0.0123 |
| 2012 | -0.0261 | 0.0180 | -1.45 | 0.148 | -0.0614 | 0.0092 |
| 2013 | -0.0118 | 0.0181 | -0.65 | 0.514 | -0.0473 | 0.0237 |
| 2014 | -0.0189 | 0.0180 | -1.05 | 0.293 | -0.0542 | 0.0164 |
| 2015 | -0.0193 | 0.0177 | -1.09 | 0.277 | -0.0539 | 0.0154 |
|  |  |  |  |  |  |  |
| $\overline{TMesothelioma}$ | **0.3835** | **0.1118** | **3.43** | **0.001** | **0.1643** | **0.6027** |
| $\overline{TOccupResp}$ | -1.0502 | 0.6718 | -1.56 | 0.118 | -2.3669 | 0.2664 |
| $\overline{TCopd}$ | **0.2264** | **0.0298** | **7.6** | **0** | **0.1680** | **0.2847** |
| $\overline{TischemHeart}$ | **0.0532** | **0.0109** | **4.89** | **0** | **0.0319** | **0.0746** |
| $\overline{{pm}_{2.5}}$ | **0.0139** | **0.0015** | **9.04** | **0** | **0.0109** | **0.0170** |
|  |  |  |  |  |  |  |
| $Urbanization$ |  |  |  |  |  |  |
| 2 | 0.0232 | 0.0302 | 0.77 | 0.442 | -0.0360 | 0.0824 |
| 3 | **0.1217** | **0.0301** | **4.04** | **0** | **0.0627** | **0.1807** |
| 4 | **0.1676** | **0.0285** | **5.89** | **0** | **0.1118** | **0.2234** |
| 5 | **0.3034** | **0.0311** | **9.77** | **0** | **0.2425** | **0.3643** |
|  |  |  |  |  |  |  |
| $Deprivation$ |  |  |  |  |  |  |
| 2 | 0.0177 | 0.0165 | 1.07 | 0.285 | -0.0147 | 0.0501 |
| 3 | -0.0153 | 0.0185 | -0.83 | 0.406 | -0.0515 | 0.0209 |
| 4 | **-0.0946** | **0.0256** | **-3.7** | **0** | **-0.1448** | **-0.0445** |
| 5 | **-0.1901** | **0.0271** | **-7.02** | **0** | **-0.2432** | **-0.1370** |
|  |  |  |  |  |  |  |
| $Radon$ |  |  |  |  |  |  |
| [50,100) | -0.0027 | 0.0180 | -0.15 | 0.88 | -0.0380 | 0.0325 |
| [100,150) | **0.0974** | **0.0223** | **4.37** | **0** | **0.0537** | **0.1411** |
| [150,300) | **0.0998** | **0.0288** | **3.47** | **0.001** | **0.0434** | **0.1562** |
| [300+) | 0.1549 | 0.0962 | 1.61 | 0.107 | -0.0336 | 0.3434 |
